# Measuring the Quality of Species List Contents

**DOI:** 10.1093/biosci/biaf191

**Published:** 2026-01-28

**Authors:** Thomas Pape, Richard L Pyle, Olaf Bánki, Saroj K Barik, Alex J Berryman, Patrice Bouchard, John Buckeridge, Les Christidis, María Marta Cigliano, Stijn Conix, Haylee Crawford-Weaver, Peter Paul van Dijk, Markus Döring, Neal Evenhuis, Craig Hilton-Taylor, Donald Hobern, Claire Johnston, Ronell R Klopper, Andreas Kroh, Marianne Le Roux, Aaron M Lien, Lauren Raz, Scott Thomson, Leen Vandepitte, Frank E Zachos, Stephen T Garnett

**Affiliations:** Natural History Museum of Denmark, University of Copenhagen, Copenhagen, Denmark; Department of Natural Sciences, Bernice Pauahi Bishop Museum, Honolulu, Hawaii 96817, USA; Catalogue of Life, Catalogue of Life, Secretariat, Pampuslaan 59, 1382 JM Weesp, Amsterdam, The Netherlands; Department of Botany, North-Eastern Hill University, Shillong, Meghalaya, India; BirdLife International, Cambridge, United Kingdom; Canadian National Collection of Insects, Arachnids and Nematodes, Agriculture and Agri-Food Canada, 960 Carling Avenue, Ottawa, Ontario K1A 0C6, Canada; Earth and Oceanic Systems Group, RMIT University, Melbourne, VIC 3001, Australia; Southern Cross University, Coffs Harbour, New South Wales 2450, Australia; Museo de La Plata, Centro de Estudios Parasitológicos y de Vectores - Consejo Nacional de Investigaciones Científicas y Técnicas, Universidad Nacional de La Plata, La Plata, Argentina; Institut Supérieur de Philosophie, Université Catholique de Louvain, Louvain-La-Neuve 1348, Belgium; Department of Climate Change, Energy, the Environment and Water, GPO Box 3090, Canberra ACT 2601, Australia; Re: wild, P.O.Box 129, Austin, TX 78767, USA; Catalogue of Life/Global Biodiversity Information Facility, Universitetsparken 15, DK-2100, Copenhagen Ø, Denmark; Department of Natural Sciences, Bernice Pauahi Bishop Museum, Honolulu, Hawaii 96817, USA; International Union for Conservation of Nature Red List Unit, David Attenborough Building, Pembroke Street, Cambridge CB2 83QZ, UK; Catalogue of Life, Catalogue of Life, Secretariat, Pampuslaan 59, 1382 JM Weesp, Amsterdam, The Netherlands; Research Institute for the Environment and Livelihoods, Charles Darwin University, Casuarina, NT 0909, Australia; Foundational Biodiversity Sciences Division, South African National Biodiversity Institute, Pretoria, South Africa; H.G.W.J. Schweickerdt Herbarium, Department of Plant and Soil Sciences, University of Pretoria, South Africa; Natural History Museum Vienna, Vienna, Austria; Foundational Biodiversity Sciences Division, South African National Biodiversity Institute, Pretoria, South Africa; Department of Botany and Plant Biotechnology, University of Johannesburg, Johannesburg, South Africa; Research Institute for the Environment and Livelihoods, Charles Darwin University, Casuarina, NT 0909, Australia; School of Natural Resources and the Environment, University of Arizona, Tucson, AZ, USA; Instituto de Ciencias Naturales, Universidad Nacional de Colombia, Bogotá, D.C., Colombia; Research Institute for the Environment and Livelihoods, Charles Darwin University, Casuarina, NT 0909, Australia; Centro de Estudos dos Quelônios da Amazônia, Manaus, Brazil; Flanders Marine Institute, Jacobsenstraat 1, Oostende 8400, Belgium; Research Institute for the Environment and Livelihoods, Charles Darwin University, Casuarina, NT 0909, Australia; Natural History Museum Vienna, Vienna, Austria; Department of Evolutionary Biology, University of Vienna, Vienna, Austria; Department of Genetics, University of the Free State, Bloemfontein, South Africa; Research Institute for the Environment and Livelihoods, Charles Darwin University, Casuarina, NT 0909, Australia

**Keywords:** taxonomy, species list scores, indicators, Catalogue of Life, standards

## Abstract

Taxonomic lists, usually of species, have many functions. However, there is currently no reliable and convenient way to determine whether a list contains the information that a user requires other than by reading the list in detail. We therefore developed 24 indicators to characterise list contents. The indicators aim to describe the extent to which the scored list covers their intended class of organisms, the quality of their taxonomic scholarship, and additional information they provide for each taxon. We tested the indicators on 16 lists drawn from a wide range of vertebrates, invertebrates, and plants. A list content score was derived from individual indicator scores after they had been weighted to reflect the preferences that taxonomists had expressed in a global survey. The indicators aim to help list creators provide the details taxonomists and list users consider important. We expect indicators to be refined after public debate..

Lists of species or other taxonomic ranks have many roles in science and human society, underpinning numerous aspects of biology, ecology, conservation, and other societal priorities. They are also key instruments for communicating about biodiversity (Palmer et al. 1995, Costello and Wieczorek 2014, Ruggiero et al. 2015). We define the term *species lists* to refer to taxonomic lists of scientific names formed in accordance with standard methods of scientific nomenclature, which represent taxonomic entities important for communication about biodiversity. Although most species lists refer to taxonomic entities labeled at the rank of species, they often include entities labeled with names for taxonomic ranks at both higher (e.g., genera) and lower (e.g., subspecies) ranks.

Poorly constructed lists, and situations where conflicting lists are followed by different people, cause confusion among list users and can have negative social consequences (Palmer et al. 1995, Palmer and Richardson 2012, Thomson et al. 2021). Likewise, the uncritical use of lists created by different taxonomists (who can differ in their views of what constitutes a species) may bias analyses in evolutionary and comparative biology (e.g., Palmer et al. 2008, Faurby et al. 2016) and prevent the integration of data needed for global conservation initiatives (Feng et al. 2022, Sandall et al. 2023). Although it is important that taxonomists have the freedom to create as many lists for a group as they see fit, the potential users of a list need to have access to information that will allow them to assess the qualities of a list if they are to know whether it will suit their needs. An initial set of recommendations of what information should be associated with each entry on a good species list was outlined by Pyle and colleagues (2021) and echoed earlier work by Palmer and colleagues (1995). These authors pointed out that it must be possible to understand not only what is represented by each entry on a list but also a list’s accuracy, consistency, uncertainties, and the procedures used to create, update, and maintain it. In all, Pyle and colleagues (2021) identified 31 details (supplemental table S1) that they felt should be considered in list creation, many of which correspond to the 32 minimal and 24 desirable qualities that Palmer and colleagues (1995) considered should be included in regional floras.

The relative importance of some of these different list details was assessed by over 1000 taxonomists and taxonomic list users in a recent survey (Lien et al. 2023). The respondents preferred lists containing unique species identifiers (i.e., unique numbers or codes beyond the names and labels themselves that are linked to the names), a hierarchical taxonomic classification from the rank of species up, information on the authors of the treatment and nomenclature, and statements about uncertainty in taxonomic classification and status. They were less enthusiastic about vernacular names and illustrations. A key concern for the respondents was that many existing lists do not allow them to judge the provenance of the data included.

For users of lists and for taxonomists, understanding the quality of a list affects the decision about whether it is adequate for the purpose required. For some users, shortcomings in, for example, the completeness of the review of synonyms (and, therefore, an indication of the taxonomic scope or taxon concept represented by each item in a list) can render a list unusable (e.g., Franz et al. 2008, 2016). Others require only that a list provide comprehensive coverage of species in a family (e.g., IUCN 2024). Such information, however, is rarely provided as part of a list’s metadata; only detailed analysis of a list can reveal its shortcomings. This points to a need for a tool that can succinctly describe the quality of a list so that users can understand its strengths and weaknesses.

We present such a tool in the present article, providing a system for scoring each of the list details described by Pyle and colleagues (2021) (supplemental table S1). We have two specific aims: One is to create a tool that can help improve the practice of list creation, guiding those who create lists to improve their practice, particularly if they wish their lists to be widely used. The other is to provide a form of quality assurance for the users of lists who lack the background knowledge of taxonomy needed to judge the adequacy of the list details.

We first developed a set of quality indicators. To do so, we followed four steps commonly followed for developing composite indices: indicator selection, normalization, weighting, and aggregation (Gómez-Limón and Sanchez-Fernandez 2010). Scores for individual indicators can be used to assess whether a given list is fit for purpose in a particular context. We then aggregated the scores into a single metric that aims to characterize the overall quality of the list. To ensure our draft indicators are practical, we tested the indicators on a diverse set of 16 lists (table 1).

**Table 1.** Taxonomic lists used to test indicators.

Grouping	Listed group	Name of list (no. of names/accepted species)	No. of names	No. of accepted species	Reference
Plants	Conifers	Conifer database	8,408	3,564	Farjon et al. (2015)
	Conifers	Conifers (subclasses Pinidae and Cupressidae)	3,708	717	World Flora Online (2024a,b)
	Legumes	World Checklist of Vascular Plants: Fabaceae	89,452	22,557	Legume Phylogeny Working Group (2024)
	Bryobionta, Vascular plants	South African National Plant Checklist	63,433	25,599	SANBI (2024)
Invertebrates	Echinoderms	World Echinoidea Database	9,905	4,263	Kroh and Mooi (2025)
	Orthopterans	Orthoptera Species File	76,122	30,319	Cigliano et al. (2024)
	Flies	Systema Dipterorum	210,993	174,823	Evenhuis and Pape (eds) (2024)
	Sesiid moths	Checklist of the Sesiidae of the world	3,742	1,582	Pühringer and Kallies (2004)
	Beetles	Type genera of Coleoptera	5,304	4,780	Bouchard et al. (2024)
Vertebrates	Fish	Fishbase	139,785	36,001	Froese and Pauly (2024)
	Fish	Eschmeyer’s Catalog of Fishes	62,306	37,140	Fricke et al. (2025)
	Turtles	Turtles of the World	1,488	486	Turtle Taxonomy Working Group (2021)
	Birds	BirdLife International Illustrated Checklist of The Birds of The World	11,120	11,120	Del Hoyo and Collar (2014, 2016)
Fossils	Fossil barnacles	Cirripedia	414	414	Chan et al. (2021)
	Fossil echinoderms	World Echinoidea database	5,443	3,250	Kroh and Mooi (2025)
	Fossil turtles	Pleistocene and Holocene chelonians	220	112	Turtle Extinction Working Group (2015)

Indicator scores can be used to provide an overview of list quality to prospective list users and by the creators of lists to identify potential improvements that could increase a list's utility and broaden its appeal. This approach can be seen as expanding on the metrics used to characterize regional floras in the United States (Palmer and Richardson 2012). However, where Palmer and Richardson (2012) described a set of minimal standards, we provide a graded set of scores leading up to an aspirational ideal. Given the concerns of Espeland and Stevens (2008) that quality metrics can be used to control the behaviour of people whose work is being measured, we have also tried to create a tool that is simple to apply and that measures features of lists that the community of list creators, taxonomists, and list users consider important and relevant to a list's quality. We emphasise that we do not see this as a final set of polished indicators. Rather, our purpose is to provide the taxonomic community with a methodology that can be refined further after public debate.

## Indicator selection

Recommendations regarding what a species list ought to contain were outlined by Pyle and colleagues (2021) as a set of list details (supplemental table S1; see also Palmer et al. 1995, Hobern et al. 2021). Additional aspects of list quality were identified by creators of the 16 case study lists (table 2). A total of 24 indicators of list content quality were articulated, with the language iteratively refined to remove ambiguities and increase precision and clarity.

**Table 2.** Indicators for assessing the content quality of species lists (based largely on Pyle et al. 2021), an example of a statement that would be published with the list that would represent an ideal state for that indicator, and a weight representing the importance given to the indicator by taxonomists and users of taxonomy (derived from Lien et al. 2023).

List indicator	Example of statements in list introduction that would satisfy a score of 5	Weight (out of 10)
Scope: the extent to which a list articulates its taxonomic and geographic and/or ecological coverage.	The list includes species (or other specified taxonomic unit [specify unit]) in the following taxonomic group [specify group] from the entire world/the following geographic regions/habitats: [specify region/habitat].	8.2
Completeness: the extent to which a list covers all known extant and recently extinct (since 1500 CE) taxa in specified group.	The list covers all known extant and recently extinct (since 1500 CE) taxa within the scope of [specified group].	8.7
Recently extinct taxa: whether a list includes taxa known to have become extinct since 1500 CE, if any.	All taxa known to have become extinct since 1500 CE are included and a source for extinction listing cited. OR No taxa in group are known to have become extinct since 1500 CE.	7.9
Fossils: whether taxa that became extinct before 1500 CE are included.	No data on fossils is provided. OR The following data on fossils has been included: [describe type and comprehensiveness of data provided on fossils]. OR No taxon is known from fossils.	5.4
Non-code-regulated names: the extent to which the list includes non-code-regulated names (provisional names, hybrids, etc.), if such names are widely used and have professional applications within the group (e.g., angiosperms, vertebrates).	Non-code-regulated names (provisional names, hybrids, etc.) are [provided or not provided] in addition to code-regulated names [because non-code-regulated names are not widely used in the group of organisms].	3.0
Prelisting review: an indication of the basis for the minimum quality of evidence needed before status is accepted.	Newly described or revised taxa are included unconditionally/provisionally until [state conditions of full inclusion]/excluded until [state conditions of full inclusion].	8.2
Nomenclatural code: whether the nomenclatural code (or multiple codes for ambiregnal taxa) is specified.	The list is consistent with the following nomenclatural code or code[s]: [name of relevant code [with the following exceptions: [name exceptions]].	6.6
Classification detail: the extent to which detail is provided of taxonomy appropriate to the scope other than genus and species (i.e., subspecies, family, order, class, phylum, domain).	The list includes as much classification detail of the taxonomy (other than genus and species) as is available and appropriate. This includes: [specify detail provided].	8.7
Unique, persistent identifier: whether each name is associated with a unique, persistent identifier.	Every name on the list is associated with a unique, persistent identifier.	8.5
Nomenclatural authority: whether names are associated with an author (and date of publication for taxonomic groups where this is common practice).	Every name listed has an author/authors associated with it [and, if appropriate for the group, dates of publication (year or month plus year depending on best practice within group)].	9.5
Treatment authority: whether the accepted status of names on a list is associated with a taxonomic treatment in which the acceptance status is represented.	Every name in the list includes reference to the taxonomic treatment in which its status is represented as accepted. OR The list represents a baseline list for acceptance of the group of organisms listed.	8.7
Source of name: whether there is a link or textual description of the database or working group that provided the taxonomic and nomenclatural entry to the global list, with some minimal amount of associated properties of the source, or a link to source metadata.	Information is provided on the source of every taxonomic and nomenclatural entry that includes a link/textual description of the database/working group that provided the taxonomic and nomenclatural entry to the global list, with information on the properties of the source/a link to source metadata.	8.2
Original ranks and combinations: whether information is provided on the combination of names used in the basionym (if applicable) or the original combination, synonyms, or if the taxonomic rank has been changed.	Information is provided for every listed taxon on the basionym combination, or the original rank for names for taxa treated as subgenera/subspecies that were originally established as full genera/species, and vice versa.	9.1
Original literature citation: whether the full literature is cited on the original establishment of the name (e.g. the basionym).	The full literature on the original establishment of the name (e.g. the basionym) is provided for all entries.	9.1
Citation completeness: whether the full literature on each combination subsequent to the basionym is also cited (for disciplines where this is common practice; e.g., botany, mycology).	All listed names include full citations for combinations. OR Citations for combinations are not included because this is not common practice in discipline.	8.2
Current status literature citation: whether the full literature is cited on the current status of the name (e.g., combination, synonymy, spelling).	The full literature on the current status of every listed name is provided (i.e., all authors, full title, year, journal, volume, pages) including for combinations, synonymy, and spelling.	8.2
Homotypic (i.e., objective or nomenclatural) synonyms: the extent to which homotypic synonyms (names based on the same type) are provided.	The list includes all known homotypic synonyms (i.e., subsequent combinations, alternate spellings, alternate ranks, replacement names, and orthographic variants) known to the list creator.	10.0
Heterotypic (i.e., subjective or taxonomic) synonyms: the extent to which heterotypic synonyms (names based on different types) are provided.	The list includes all known heterotypic synonyms known to the list creator.	10.0
Confidence in taxonomic rank and placement: an articulation of the level of acceptance of the taxonomy among relevant taxonomists.	Confidence in taxonomic rank and placement is described as follows: [description of approach taken to articulating the level of acceptance of the taxonomy].	8.2
Documentation of change: the extent to which changes to a list from previous versions are documented.	Complete information is provided on list version history so that there is a data audit trail for every change to the database.	8.8
Geographical distribution: whether distributional information has been included.^a^	The following distributional information has been included: [describe type and comprehensiveness of distributional information provided; can include none even though available]. OR No distributional information is available].	9.9
Images: whether imagery has been included.^a^	The following imagery has been included: [describe type and comprehensiveness of imagery provided; can include none even though available]. OR Imagery is not available.	8.7
Genetic data: whether genetic data has been included.^a^	The following genetic data has been included: [describe type and comprehensiveness of genetic data provided; can include none even though available]. OR No genetic information is available.	8.2
Additional information: whether additional information on taxon biology has been included.^a^	The following additional information has been included: [describe type and comprehensiveness of additional information provided, if any].	7.1

^a^Desirable but inessential indicators.

Indicators were placed in one of three groups. One set describes aspects of the breadth of taxonomic components described in a list. The second set articulates the detail included within the list. The third set covers a set of features of a list that, although they are desirable features that might improve a list’s functionality, are not critical to its quality (table 2). For each indicator, we identified the facet of a list to which it applied, the ideal state and, when possible, the weight attached to the indicator based on Lien and colleagues (2023; supplemental table S2). We then identified the set of intermediary suboptimal states for each indicator (table 3).

**Table 3.** Scoring protocols for indicators for assessing the content quality of species lists. "na" denotes scores for which no intermediary indicator was identified.

Indicator	Contribution to total score %	Score					
		0	1	2	3	4	5
Scope	4.1	Taxonomic and geographic/ecological scope potentially ambiguous	na	na	na	na	Taxonomic and geographic/ecological scope fully articulated
Completeness	4.4	Current status citation not provided	List includes less than 50% of known target taxa	List includes 50%–74% of known target taxa	List includes 75%–89% of known target taxa	List includes 90%–99% of known target taxa	List includes all known target taxa
Recently extinct taxa	4.0	Recently extinct taxa excluded	Some recently extinct taxa included but no information source cited for taxa listed as extinct	Some recently extinct taxa included but no information source cited for taxa listed as extinct	na	All recently extinct taxa included but no information source cited for taxa listed as extinct	All recently extinct taxa included. Information source cited for taxa listed as extinct OR no listed taxa are thought to have become extinct since 1500 CE
Fossils	2.8	No information provided on whether taxa that became extinct before 1500 CE included	na	na	Statement that taxa that became extinct before 1500 CE included, but no further detail	na	Statement that taxa that became extinct before 1500 CE included with detail about coverage OR statement that none included
Non-code-regulated names	1.5	No information on whether non-code-regulated names included, even if they are widely used in the group	na	na	Non-code-regulated names included but no further information on which names or completeness of coverage	na	Non-code-regulated names not used in group OR explicit statement that non-code-regulated names not included OR statement describing the extent to which they are included (type, completeness)
Inclusion (contribution to score)	4.1	No information provided on inclusion criteria	na	na	na	na	Detailed description of inclusion criteria
Nomenclatural code	3.4	Nomenclatural code not specified	na	na	na	Nomenclatural code is self-evident	Nomenclatural code specified
Classification detail	4.4	Genus and species the only level of classification provided	List omits four levels of classification detail other than genus and species even though available and appropriate	List omits three levels of classification detail other than genus and species even though available and appropriate	List omits two levels of classification detail other than genus and species even though available and appropriate	List omits one level of classification detail other than genus and species even though available and appropriate	List includes as much classification detail other than genus and species as available and appropriate
Unique, persistent identifiers	4.3	No unique, persistent identifiers provided	Unique, persistent identifiers provided for 1%–24% of entries on list	Unique, persistent identifiers provided for 25%–49% of entries on list	Unique, persistent identifiers provided for 50%–74% of entries on list	Unique, persistent identifiers provided for 75%–99% of entries on list	Every name has a unique, persistent identifier
Nomenclatural authority	4.8	No name has author (or dates)	Some (but not all) names have author (or date if date inclusion is normal practice)	na	Many or most names have author (and date if date inclusion is normal practice)	na	Every name has author[s] (and precise dates of publication (year or month plus year depending on best practice within group) if date inclusion is normal practice)
Treatment authority	4.4	No information provided on the taxonomic treatment in which the status of each taxon is represented as accepted	Some (but not all) names include reference to the taxonomic treatment in which their status is represented as accepted	na	Treatment author only included when there have been recent changes or changes are proposed	na	Every name includes reference to the taxonomic treatment in which its status is represented as accepted
Source of name	4.1	No information provided on source of the entry	Some but not all names have taxonomic and nomenclatural information provided on source of the entry	na	na	na	Information provided on source of every taxonomic and nomenclatural entry
Original ranks and combinations	4.6	No information provided on the basionym combination, or whether the taxonomic rank has changed	Information is provided for some taxa on the basionym combination, or whether the taxonomic rank has changed	na	na	na	Information is provided for every taxon on the basionym combination or whether the taxonomic rank has changed
Original literature citation	4.6	Original publication which established the exact combination not cited	Less than half of entries cite original publication which established the exact combination	50%–90% of entries cite the original publication which established the exact combination in part (e.g., only first author, year)	50%–90% of entries cite the original publication which established the exact combination in full (i.e., all authors, year, journal, volume, pages)	91%–100% of entries cite the original publication which established the exact combination in part (e.g., only first author, year)	91%–100% of entries cite the original publication which established the exact combination in full (i.e., all authors, year, journal, volume, pages)
Citation completeness	4.1	No citation provided for combinations (for disciplines where this is common practice)	Some but not all names include partial citations for combinations (for disciplines where this is common practice)	Some but not all names include full citations for combinations (for disciplines where this is common practice)	na	Every name includes partial citations for combinations (for disciplines where this is common practice)	Every name includes full citations for combinations (for disciplines where this is common practice) OR not common practice in discipline
Current status literature citation	4.1	Current status citation not provided	Some but not all entries have the partial current status citation provided (e.g., only first author, year)	Some but not all entries have the partial current status citation provided (e.g., only first author, year, journal, volume, pages)	Some but not all entries have the full current status citation (e.g., complete literature citation, with all authors, full title, etc.)	Partial current status citation provided for every entry	Full current status citation provided for every entry
Homotypic synonyms	5.1	No homotypic synonyms provided	Less than 50% homotypic synonyms known to the list creatorprovided	50%–74% homotypic synonyms provided	75%–89% homotypic synonyms provided	90%–99% homotypic synonyms provided	Every known homotypic synonym known to the list creator is provided
Heterotypic synonyms	5.1	No heterotypic synonyms provided	Less than 50% heterotypic synonyms known to the list creator provided	50%–74% heterotypic synonyms provided	75%–89% heterotypic synonyms provided	90%–99% heterotypic synonyms provided	Every known heterotypic synonyms known to the list creator is provided
Confidence in taxonomic rank and placement	4.1	No information provided on confidence in taxonomic rank and placement of taxa	na	na	na	na	Description of how variation in confidence in taxonomic rank and placement of taxa has been accommodated
Documentation of change	4.5	No information provided on the list version history	Limited information (e.g., version number and other very basic information) provided on list version history	na	Partial version history, or version history can be reconstructed (but not automatically)	na	Complete information provided on list version history (i.e., data audit trail with every change to the database logged)
Geographical distribution	5.0	No information provided on whether distributional information included	Distributional information not included even though available	na	Distributional information included but no detail on type or completeness	na	Distributional information included with detail on type or completeness OR statement that none available
Images	4.4	No information provided on whether imagery included	Imagery not included even though available	na	Imagery included but no further detail on type, completeness or coverage	na	Imagery included with detail on type and completeness OR statement that none available
Genetic data	4.1	No information provided on whether genetic data included	Genetic data not included even though available	na	Genetic data included but no detail on type, completeness or coverage	na	Genetic data included with detail on type and completeness/statement that none available
Additional information	3.6	No information provided on whether additional biological information included	No additional information included	na	Additional biological information included but no further detail on type or completeness	na	Additional biological information provided with detail on type and completeness

### List breadth indicators

Users of a list first need to know what they can expect to find should they consult the list. We developed five indicators that characterize the components of a list. These included a description of the group of organisms being listed, the comprehensiveness of the list for that group, whether the list includes any recently extinct taxa or organisms with non-code-regulated names, and the criteria for inclusion of new taxonomic hypotheses.

Explicit articulation of the scope of a list is essential for users, with utility being greatest for lists that aim for comprehensive coverage of taxa commonly managed as a group because of taxonomic affinity or historical contingency. Most lists will be of species (and taxa of subordinate ranks), but lists of other taxonomic units may also be valuable (e.g., genera: Bouchard et al. 2024). Some lists will be of regions or habitats with taxa from many groups, others will be global from a single group. The highest scores were given to lists that fully explained the taxonomic, geographical, or ecological bounds of the list. Palmer and colleagues (1995) considered this the most important aspect of a list, but Palmer and Richardson (2012) found it to be the feature most likely to be deficient in regional floras. They also suggested that the title be explicit about the list's scope, but compliance with digital formats may constrain title formats, so this is excluded from the present conditions for high scores.

Although it was not considered by Pyle and colleagues (2021) because they focused on a single global species list, the comprehensiveness of a list is an important consideration for the partial lists that together will make up a single global species list. Although nearly all lists are likely to be incomplete because additional species may be newly described or found after being overlooked in older literature, the lists receiving the highest score included every species (or other taxonomic unit) in the taxonomic group defined by the scope of the list and known at the time of the list's preparation.

The decision on whether to include extinct taxa is related to completeness. The IUCN Red List includes all taxa that have become extinct since 1500 CE (Pyle et al. 2021, detail 4). Taxa that are assumed to be extinct may be rediscovered, sometimes long after extinction is suspected, so that, if a taxon is to be noted as extinct on a list, the standard of evidence for extinction also warrants attention, given the implications of such listing (Martin et al. 2023). We gave the highest values to lists of extant species that also included any taxa known or suspected to have become extinct since 1500 CE.

Many taxa that became extinct before 1500 CE are likely to be known only from fossils (Pyle et al. 2021, detail 4). Some lists consist entirely of names for such taxa. Other lists ignore taxa known only from fossils even though such taxa have been described for that group. For many groups, no fossils are known. Lists may also contain both extinct taxa known only from fossils and extant taxa, which can be relevant if, for example, extant and extinct taxa occur in the same deposits. We gave the highest score to lists that were explicit about their inclusion of fossils, with no judgement on the merits of doing so.

List compilers need to decide whether to include regulated but non-Linnean nomenclature systems (Pyle et al. 2021, detail 3). These include those related to plant cultivars, some trade names, DNA sequence codes, and provisional temporary or tag names that are used for taxa as an alternative to, or before, formally naming them under the codes of nomenclature (Winston 2018, Huber et al. 2024). This includes higher unranked taxon names, such as Bilateria, which are important for organizing lists even if they are not strictly code-regulated. Also included in this indicator is the potential listing of unnamed nodes of hypothesized phylogenies (Pyle et al. 2021, detail 5). For many lists, however, these extra names are either undesirable or unavailable. Higher scores were given to lists that explained clearly what they included.

As was noted by Pyle and colleagues (2021, detail 2), list compilers need to decide which of three approaches they take to inclusion of new taxonomic hypotheses: to include, without review, all newly described or revised taxa as soon as possible after the date of publication; to list them provisionally until there is evidence they have been accepted by the taxonomic community; or to exclude them until conditions for full inclusion have been met. The indicator makes no judgement on which approach to prelisting screening is taken. Rather, the highest scores were given to lists in which the approach taken was explicit and the conditions for full acceptance, if it was provisional, were articulated.

### List detail indicators

Once taxa are included, documentation is required of the detail that accompanies each of the listed taxa, including the code under which they are named, the detail provided on their classification, the information needed to track taxonomic concepts (10 indicators), the confidence around different aspects of the listing, and the extent to which the list has changed.

Information on the code adhered to by a list (or more than one code for ambiregnal species; Pyle et al. 2021, detail 11) has implications for both governance (i.e., does a list follow the rules of a code; see Garnett et al. 2025) and contents (with which rules might a list be expected to comply). The highest scores were given to lists in which there was explicit articulation of the code used (or multiple codes for lists of taxa falling under multiple codes; e.g., national lists of plants and animals).

Pyle and colleagues (2021, details 1 and 17 in supplemental table S1) argued that lists of species names should also include taxonomic ranks above and below the species so that list contents can be classified using defensible biological criteria. Not all newly discovered forms of life can necessarily be placed into a genus or other taxonomic category immediately, but that does not necessarily preclude their reporting in a list. Scores for this indicator increased with the number of higher taxon groupings above the rank of genus (i.e., family, order, class, phylum, domain) or below the rank of species (i.e., subspecies, varieties) because the more detail that is provided, the more readily relationships and patterns can be investigated.

Globally unique identifiers for names that allow metadata to be unambiguously and persistently resolved or retrieved and that are robust and consistent with best practice biodiversity information standards (Pyle et al. 2021, detail 6; see also detail 8 on labeling and Pyle and Michel 2008, Penev et al. 2016, Wrankmore et al. 2022) are increasingly being included in lists. The highest scores were given to lists in which such identifiers were applied universally.

Nomenclatural authorship of the scientific name formatted in a form recommended by the relevant nomenclatural code or widely applied in the relevant community (Pyle et al. 2021, detail 9) was considered essential by the respondents to Lien and colleagues' (2023) survey. A less than perfect score for this indicator may render a list unacceptable to some users. Lists received the highest scores if nomenclatural authorship was available for every name (especially for species but also for names at higher and lower ranks).

For many lists, the list authors decide whether a taxonomic arrangement is accepted. Other lists aggregate published treatments of names. Knowing the authority relied on for the judgement about acceptance can potentially be as important as knowing the nomenclatural author (Pyle et al. 2021, detail 10), so the highest scores were applied to lists where this information was explicit.

Most lists are compiled from multiple sources (publications or databases). Higher scores were associated with more detail on the sources for names included on the list.

Lists received the highest scores if they had included all available information on the combination of names used in the basionym (if applicable) or the original combination, synonyms, or if the taxonomic status had changed (e.g., names for taxa treated as subgenera or subspecies that were originally established as full genera or species and vice versa; Pyle et al. 2021, detail 12).

The highest score was given to lists with complete bibliographic details for the publication that first established a name in a code-compliant way (Pyle et al. 2021, detail 13).

For taxon groups governed by the International Code of Nomenclature for Algae, Fungi, and Plants (ICNafp), there is a tradition for citing the full literature for each nomenclatural combination subsequent to the original combination (basionym); lists of names for such organisms (governed by ICNafp) received higher scores if this information had been provided. Lists composed exclusively of names governed by other nomenclatural codes scored 5 for this indicator.

As for the original and subsequent literature, lists with the most complete citation of the literature containing the treatment representing the current taxonomic status of a name (Pyle et al. 2021, detail 14) were given the highest score.

The listing of homotypic synonyms (alternative names but having the same type specimen), misspellings, and other orthographic variants is often essential for alignment of lists from different sources (Pyle et al. 2021, detail 16). To receive the highest score, lists had to have included a statement that a list included all homotypic synonyms known to a list's creator[s].

Listing heterotypic synonyms (alternative names with a type specimen that is different from that of the accepted name) is helpful not only for alignment with lists from different sources but also for recognizing the circumscriptions of the taxa in the list (Pyle et al. 2021, detail 16). As with homotypic synonyms, lists that included a statement that a list includes all heterotypic synonyms known to a list's creator[s] were given the highest score.

Even though the taxonomic ranks of taxa on lists and their placement within the taxonomic hierarchy have been accepted by the list creators, confidence can vary, meaning some names are more likely to be revised than others (Pyle et al. 2021, detail 15). One such system that has recently been introduced (and applied to the Felidae species list by the IUCN Cat Specialist Group) is the traffic-light system of Kitchener and colleagues (2022) that indicates different levels of certainty in support within the community with respect to recognition of felid taxa. Lists receiving the highest score described how confidence in the taxonomic rank and placement of each taxon is considered.

Lists change frequently. Lists with complete data-audit trails with every change to the database logged in a version history (Pyle et al. 2021, detail 7) were given the highest score, because such documentation can give list users certainty about list currency.

### Indicators of desirable but inessential list qualities

The information captured by the following four indicators is not essential to having a list of taxon names. However, many lists have invested heavily in acquiring and making available such resources. Their presence in a list was also considered desirable by many of the respondents to Lien and colleagues' (2023) survey. The scores imply no judgement on the data quality because the nature of the detail appropriate and available for a taxon group varies from list to list. Slightly lower scores are given to lists that state that they have data relevant to an indicator but for which users of a list have to make an extra effort to understand the nature of the data provided.

Distributional data on taxa can sometimes help define circumscription, confirm native or nonnative status, and allow extraction of geography-based lists (Pyle et al. 2021, detail 18). However, the true distribution of many species is poorly known and is often much altered. The detail of distributional information (e.g., regional versus global coverage), the format in which it is provided, and the nature of the geographical information (e.g., native versus introduced) can differ greatly among lists. List users do need to know, however, what is provided, including if there is none available.

The utility of lists can be enhanced by imagery, particularly if all taxa are associated with multiple high-quality images. Pyle and colleagues (2021, detail 19) noted that imagery might be provided by linked specialist image services. Ideally, list users should know the type and comprehensiveness of the imagery provided, even if that is none.

Although Pyle and colleagues (2021) acknowledged that taxonomic circumscriptions may include genetic data among the diagnostic characters, they did not specifically consider genetic sequences as a component of list quality. However, there is increasing recognition of the benefits of linking genetic information from initiatives such as the Barcode of Life (2024) and the National Center for Biotechnology Information (NCBI 2025) to species names (Hubert and Hanner 2015, Cox et al. 2025). The highest scores were given to lists that describe the type and comprehensiveness of the genetic data made available, even if that is none.

Detailed information is desirable rather than essential for list use (Pyle et al. 2021, detail 20), but some is likely to be available for all forms of life, and the respondents to Lien and colleagues' (2023) survey considered that provision of any additional information to be desirable. The highest scores were given to lists that described in detail the type of additional information provided, even if that is none.

### Excluded details

Pyle and colleagues (2021, detail 21) proposed that vernacular names should not be presented on global species lists except for items represented by an informal name. Also, although over half of the respondents to the survey of Lien and colleagues (2023) considered vernacular names important, this was the least preferred of the content options considered. All names to some extent reflect the power structures, political priorities, and world views of those who impose them (Gramsci 1982). The codes represent one worldview (Sinclair 2020), albeit one to which all Linnean taxonomy subscribes (Minelli 2020). Vernacular names reflect many other worldviews and are often highly contentious (Phipps 2019). They can be language or region specific or very broadly applied to numerous taxa, making it impossible to assess the merit of whether to include them or not or, if they are included, the merit of individual names.

A further 8 of the 31 details that Pyle and colleagues (2021; supplemental table S1) considered important were excluded from the indicators of list content quality because they relate primarily to the governance of list creation and maintenance rather than to the contents of lists. All are covered in Garnett and colleagues (2025).

## List content quality index

Index creation has three steps: normalisation, weighting and aggregation.

### Normalization

Each indicator had a minimum score of 0 for lists that failed to capture any aspect of the indicator’s intent and a maximum score of 5 if a list was considered to meet the highest state possible for that indicator. Having the same maximum score ensured that the measures for different indicators were commensurate with each other (Saisana and Tarantola 2002). Between the best and worst states, a series of up to four intermediary steps were identified with the conceptual difference between the steps approximately similar in size in terms of the information needed to move between each step, although they cannot be considered continuous variables. Refinement of the indicator wording followed the same iterative process used for the indicators themselves.

### Weighting

The relative importance of 15 of the desirable list details of Pyle and colleagues (2021; supplemental table S1) was assessed by the 1134 respondents to Lien and colleagues (2023; supplemental table S3) survey. We used these results to weight the different indicators (table 2), assuming they reflected the views of the broader taxonomic community. Initially, all preferences were normalized to a score out of 10 (i.e., each weight was multiplied by 10 and divided by the maximum weight, which therefore had a normalized score of 10.0). For indicators identified as important by the list creators during research for this article but that were not included in Lien and colleagues (2023), we used an average of all the indicators that had been weighted.

### Aggregation

To create an index of list content quality, scores were combined using linear (additive) aggregation, by summing the weighted indicators (Dobbie and Dail 2013). Although this means that one high-scoring indicator score will tend to compensate for several poor scores to a greater extent than with other aggregation approaches (Munda and Nardo 2005), the method is simple, readily understood, and the value of the overall score for an individual list matters less than the trends over time and differences between lists. The index, *C*, was calculated as follows:


(1) \begin{eqnarray*}
C = \frac{{100 \times \sum\nolimits_{{I_1}}^{{I_n}} {({S_i} \times {W_I})} }}{{\sum\nolimits_{{I_1}}^{{I_n}} {(\max \,{S_i} \times {W_i})} }},
\end{eqnarray*}

where *S* is the list contents quality score for each indicator, *i*, with *W_i_* being the weighting applied to *S* for indicator *i* as described above, *n* the number of indicators, and max*S_i_* the highest possible score of *S* (5 in all cases).

If some indicators did not apply to a list, as was the case with lists of fossils and one of genera but not species, indicator values were omitted from both the numerator and denominator of *C* so the final result could be compared with values of *C* for lists with a complete set of indicator scores.

### Scoring process

For the process of testing the clarity of the language used to describe the indicators and the steps between worst and best scores, assessments were conducted by people who were closely connected with list creation, all of whom were invited to be coauthors. Each list was scored during individual interviews between list creators and one of the authors (STG). This approach was taken for two reasons: so that the list creators could clarify the context for some of the questions and to increase clarity and precision in the language. This was particularly important in the earliest stages of list evaluation, when some elements of the process were unclear. As a result, some list scores were adjusted during the finalization of the present article as revised indicators were revisited.

## Examples of indicator application

**Figure 1.**
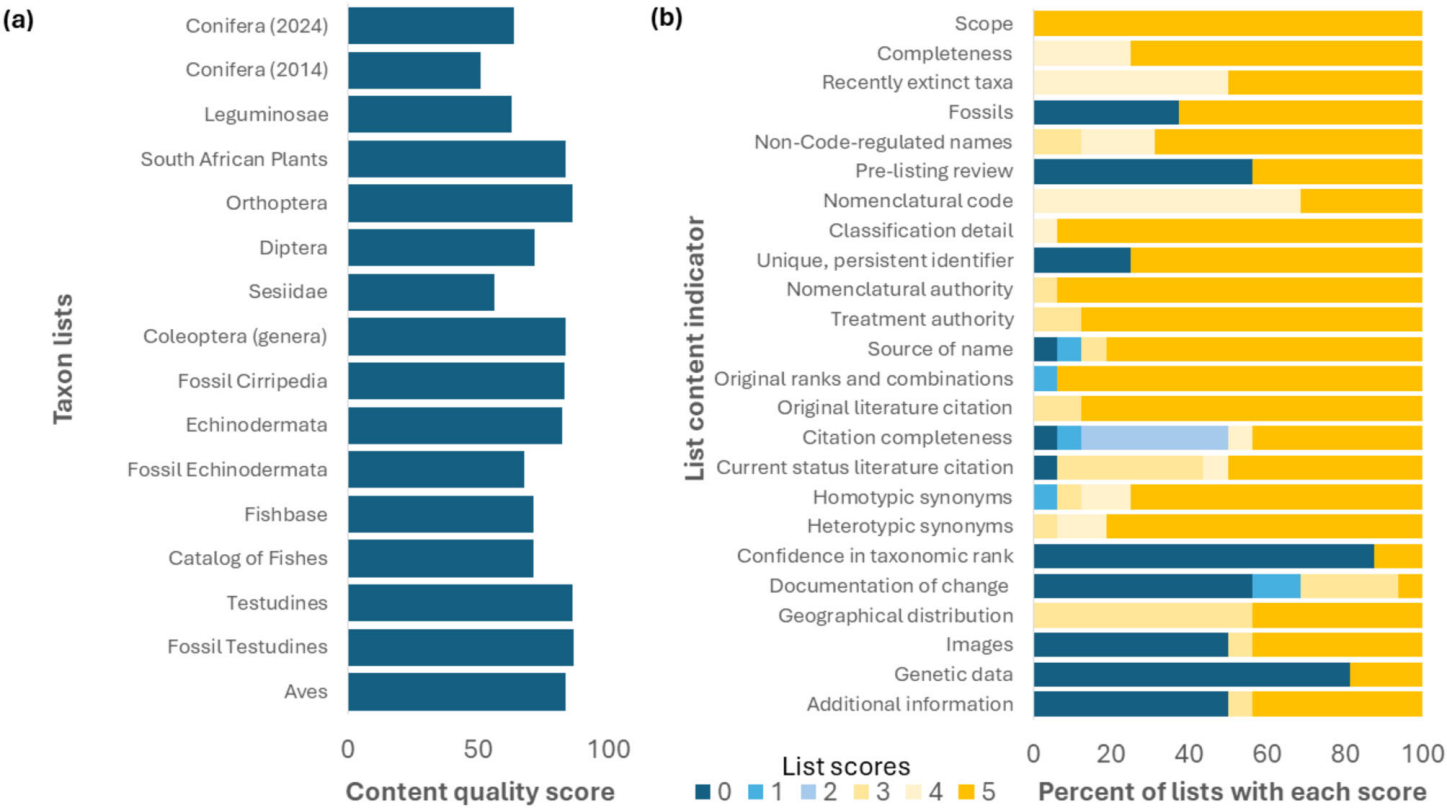
Scores from measuring the content quality of 16 taxon lists. (a) List content score based on the sum of scores for 24 indicators weighted by importance and normalized to have a maximum of 100. (b) The percentage of lists in each score category from 0 (low) to 5 (high) for each of the 24 indicators of list content quality.

The indicators were tested on 16 lists (table 2). Two lists (extant and fossil turtles) were assessed separately by two different people with connections to the database who then came to a single view. Four lists were for two pairs of the same group prepared by different people (fishes) or at different times (conifers). Another assessment (Coleoptera) was of genera, instead of species. Three lists were for fossils only. One was not a global list but rather a national list for South African plants. The overall scores (figure 1a) ranged from 51 for conifers (2014 version) to 87 for fossil turtles. The scores for eight lists exceeded 80: South African plants, Orthoptera, Coleoptera genera, fossil Cirripedia, Echinodermata, Testudines, fossil Testudines, and Aves. The average score was 73.8 (with a standard deviation of 11.7).

There was substantial variation among the indicators (figure 1b; supplemental table S3). The median score was 5, the highest level available, for over half of the indicators (14 of 24; scope, completeness, fossils, non-code-regulated names, classification detail, unique persistent identifier, nomenclatural authority, treatment authority, source of name, original ranks and combinations, original literature citation, current status literature citation, homotypic synonyms, heterotypic synonyms), but the median was 0 for five of the indicators (prelisting review, confidence in taxonomic rank and placement, images, documentation of change, genetic data). For the remaining indicators, the median was 3 or 4 (recently extinct taxa, nomenclatural code, citation completeness, geographical distribution). The variation among lists was greatest for fossils, prelisting review, images, genetic data, and additional information, for which the list scores were fairly evenly divided between 0 and 5.

The scoring of the lists' content indicators, including discussion, took 23.1 (with a standard deviation of 5.7) minutes.

### Indicators and scores

All of the 24 indicators for list content quality are consistent with the qualities expected of good indicators (table 4; Caplice and Sheffi 1994, Franceschini et al. 2019), providing objective scores for which there is no need of subjective interpretation (Franceschini et al. 2006). All details relevant to list contents enunciated by Pyle and colleagues (2021) have been covered, as have five of those mentioned by Hobern and colleagues (2021) of particular relevance to the Catalogue of Life (i.e., comprehensiveness, scope, richness, nomenclatural consistency, metadata). These were augmented during the research process by the indicator on genetics, which was considered relevant by the contributors. Although 24 is a large number of indicators, the assessment process is not arduous, and we found that most contributors could answer questions rapidly without having to refer back to their lists.

**Table 4.** Assessment of indicator quality based on a synopsis of common characteristics of good indicators (derived from Caplice and Sheffi 1994 and Franceschini et al. 2019).

Desirable indicator quality	Performance of content quality indicators
Do the indicators capture information accurately?	The information is empirical data derived from open access data sets.
Do the indicators control for inherent errors in data collection? Are they repeatable?	The indicators consist of a set of mutually exclusive steps between the worst and best case conditions, with the language tested to remove ambiguities in interpretation.
Are the indicators using a correct type of numerical scale for the values?	All measures are ordinal with the same minimum and maximum for consistency. Ordinal differences have been deliberately calibrated to detect change in the values of interest.
Do the benefits outweigh the costs of using the indicator?	Time to assess content quality (about half an hour) is an insignificant fraction of the time taken to create the list. Costs of using the indicator cannot yet be assessed against the costs of not using it.
Will the indicator create incentives for improper or counterintuitive acts?	All ideal states were judged by list creators to be desirable; no obvious opportunities for gaming the scoring were evident.
Do the indicators use data currently available from the existing ones?	All indicators are based on primary data.
Are the indicators compatible with existing information?	All information relevant to each indicator is derived from data assembled during list creation.
Do the indicators provide a guide for an action to be taken?	The description of the ideal state for the indicator provides sufficiently explicit detail for the actions required to meet it to be apparent.
Can the indicators be compared across time, location, and organizations?	All indicators should be comparable across time, location, and between lists.
Are the indicators viewed and interpreted similarly by all affected parties?	All parties involved in the content indicators have a shared view on their meaning and intent.
Are the indicators simple and straightforward enough to be easily understood by those affected?	Iterative discussions during indicator construction aimed to remove ambiguity and vagueness from indicator descriptions.
Do the indicators include and measure all of the important components and aspects of the system?	Indicators were developed for all aspects of list content that were quantifiable and so conducive to indicator development.
Do the indicators promote coordination among relevant parties?	The indicators aim to promote mutual understanding of list content, increasing use of high-quality lists.
Are the indicators of a sufficient level of detail and precision for a decision-maker?	For list creators, detail required for an ideal score is provided. For list users, detail on content should be sufficient for decision-making.

A comparison of the component scores for the different indicators shows that most indicators have high scores, perhaps a reflection of the biased sample used to develop the indicators. However, it also reveals apparent gaps in awareness about some aspects of lists that can be important. For example, few of the lists explained the basis of acceptance of new species or revisions or the confidence concerning the taxonomic placement of taxa. This is a developing area (Pyle et al. 2021), with taxonomic uncertainty having substantial practical implications for the use of species lists (Lessa et al. 2024), not least because the assignment of taxonomic rank ultimately always requires human judgement.

Although the actual scores we have obtained from the 16 lists are likely to be biased toward high quality, given that they were self-selected by authors interested in list creation, there was a measurable improvement in list content quality from 2014 to 2024 in the conifer list assessed, a change driven largely by more complete coverage of synonyms and relevant literature. Also notable is that very high scores can be achieved, as is evidenced by the seven lists for which scores exceeded 80 out of a maximum of 100.

## Potential for automation

Productivity gains through the application of artificial intelligence are starting to become apparent in many fields (Kar et al. 2023), including in academic scholarship (Noy and Zhang 2023). The creation of a taxonomic list has traditionally been a slow and arduous process requiring assiduous attention to detail and high standards of scholarship. A variety of new tools are changing the listing landscape rapidly. In particular, the repository for taxonomic data sets, ChecklistBank (Bánki et al. 2023a, 2023b, Döring et al. 2022) has been developed which is designed to allow the assessment and improvement of the quality of digital lists by combining multiple lists from different sources, matching names where needed, and drawing on both historical and recently published literature to ensure that lists contain not only the detail they need to be considered high quality but also the currency that some disciplines are now requiring (e.g., microbiology; Millett et al. 2023). Potentially, the need to link lists to genomics and genetic functionality can also be achieved using automated processes (Millett et al. 2023).

Overall, of the 24 indicators identified, 14 can be derived from the metadata that would be provided with a list, and automatic assessment of a further 8 should be possible by using ChecklistBank to analyze list contents, although 3 do require metadata for this to be achieved (supplemental table S4). If it is desired by the list creators, automatic updating of lists based on the latest literature should also be possible using ChecklistBank. Ultimately, taxonomic judgements must be made by humans, but a great deal of the checking and quality control can now be achieved automatically.

## Purpose of publishing a draft index and scoring process

Although there is widespread support among the taxonomic community for improved governance of lists and there are widely held views on what is important in a list (Lien et al. 2023), the details of how to measure improvement over existing arrangements have not been considered by the taxonomic community, with most members of that community not yet having had the opportunity to express their views or propose alternatives. There are two possible approaches to such consultation. One would be to attempt consultation widely with draft indicators before peer-reviewed publication of a final set of metrics. Although peer-review aims to improve the quality of the published product (Ware 2008), consultation before peer review imposes a high level of trust and responsibility in a small number of reviewers at the end of the process and does not represent a best practice in community consultation (Norton and Hughes 2017).

The other approach, which we have chosen to pursue in the present article, is to ensure that, before consultation with the community, we publish an approach that meets the standards required of academic peer review. Although peer review as a process is in a state of flux (Horbach and Halffman 2018), there is broad agreement that good peer review improves science (Tennant et al. 2017). It ensures that our approach has been considered of sufficiently high quality that it merits publication. We then intend to use our published papers as the basis for consultation with the taxonomic community. We hope to achieve two aims with this approach. The first is to employ a quantitative survey such as that of Lien and colleagues (2023) to develop final weights for all indicators. The second is to provide a high-quality product on which the community, whose members will collectively have a far broader diversity of experience with lists and list creation than the small authorship group and the reviewers combined, can provide comment and have input. If the process is to be adopted by taxonomists and by major aggregators of lists such as the Catalogue of Life (Hobern et al. 2021), we need to be satisfied that as wide a range of views as possible has been canvassed.

## Potential weaknesses in the approach

In a companion article on the governance of lists (Garnett et al. 2025), we consider the dangers inherent in measurement. These include the work required to do the measuring, how it changes behavior, how it can be employed to discipline human behavior, how measurements start to be important even to those who do not want to use them, and the seductiveness of the numbers created that may or may not reflect desirable qualities of the items being measured (Espeland and Stevens 2008). For many of these qualities, there is a necessary caution about introducing measurement to governance, which is a social process deeply reliant on human judgements and values. Taxonomic lists, however, are technical documents in which high levels of accuracy are needed if they are to fulfil the purposes for which they were created.

With respect to these five matters, we have developed a set of indicators that can usually be assessed in under half an hour—not a major imposition, given the effort taken to create a list and the infrequency with which measurement needs to occur. The other four factors we do not see as negative. We have created the indicators with the intention of changing behavior. Rather than set out to discipline poor behavior, we are seeking the reverse: to reward good practice by encouraging greater use of lists that attain high content quality scores, potentially with high scores being more likely to attract or sustain investment. We also see as desirable the need to aspire to high standards among list users, so we hope the measures will be adopted widely. Finally, cognizant of the link between measurement and subsequent compliance behavior (Hauser and Katz 1998), we are taking great care, through our publication and consultation processes, to produce metrics that provide relevant and useful statistics.

It should be noted that all species lists tested in the present article are global in scope, with the single exception of the South African National Plant Checklist, and although it received a high aggregate score, further testing with other national lists is desirable, because these have somewhat different objectives and different users than global species lists. Palmer and colleagues (1995) developed processes resembling ours in many ways, including scoring that was subsequently applied to many North American regional floras (Palmer and Richardson 2012). The national GBIF node of Colombia (SiB Colombia) has also proposed its own guidelines for consolidating national species lists (Torres-Mejía et al. 2016), and there may be similar cases in other countries. Further iterations of this exercise at the national level should reveal whether it is appropriate to make changes to some indicators or their weights for use in evaluating national species lists.

Finally, the interoperability of lists in a digital environment is becoming increasingly important for list usage (Feng et al. 2022). We refer to the information within taxonomic lists and their utility in the present article. We believe that the extent to which lists can be freely downloaded by users in a format they require, or transferred among different users regardless of their informatics infrastructure, warrants its own review of standards that relate not to the quality of the information itself but to the manner in which it is made available, noting the rapid changes now occurring through the development of large language models.

## Conclusions

We have provided a multifaceted, objective set of indicators to assess a species list's quality repeatably and an index that assesses overall content quality. Based on our initial consultations with the taxonomic community (Lien et al. 2023), we believe the indicators cover all the facets of a good list identified by Pyle and colleagues (2021), as well as several others considered important by the creators of existing global lists across a wide range of organisms. We have set out to make scoring simple enough so that, in about half an hour, lists can be reviewed to assess their change in quality over time. Calculation of the standard deviation based on random values allows statistical comparison of list scores over time or between lists of the same group of organisms.

The aim of creating the index is to measure the improvement in the quality of a list's contents. For many lists, this can be achieved with little extra effort on the part of taxonomists. Other facets will take considerably more time and scholarship, although resource availability is increasing. First, however, the indicators must be subject to a phase of consultation in addition to the peer-review process that has accompanied publication in this journal.

## Funding

Support for the research has been provided by Australian Research Council Grant DP230102933. The work of Stijn Conix for this paper was funded by Fonds de la Recherche Scientifique—FNRS under grant no. T.0177.21. The work of Leen Vandepitte is funded by Research Foundation—Flanders (FWO) as part of the Belgian contribution to LifeWatch.

## Supplementary Material

biaf191_Supplemental_Files

## References

[bib1] Bánki O, Döring M, Jeppesen T, Hobern D. 2023a. Demonstration of taxonomic name data services through ChecklistBank. Biodiversity Information Science and Standards. 7: e112544.

[bib2] Bánki O, Hobern D, Döring M. 2023b. Building on the functionalities of GBIF-COL ChecklistBank. Biodiversity Information Science and Standards. 7: e111668.

[bib3] Barcode of Life . 2024. BOLD Systems, vers. 5. BOLD Systems. www.boldsystems.org.

[bib4] Bouchard P, Bousquet Y, Davies AE, Cai C. 2024. On the nomenclatural status of type genera in Coleoptera (Insecta). ZooKeys. 1194: 1–981. 38523865 10.3897/zookeys.1194.106440 PMC10955229

[bib5] Caplice C, Sheffi Y. 1994. A review and evaluation of logistics metrics. International Journal of Logistics Management. 5: 11–28.

[bib6] Chan BKK, Dreyer N, Gale AS, Glenner H, Ewers-Saucedo C, Pérez-Losada M, Kolbasov GA, Crandall KA, Høeg JT. 2021. The evolutionary diversity of barnacles, with an updated classification of fossil and living forms. Zoological Journal of the Linnean Society. 193: 789–846.

[bib7] Cigliano MM, Braun H, Eades DC, Otte D. 2024. Orthoptera Species File. https://orthoptera.speciesfile.org

[bib8] Costello MJ, Wieczorek J. 2014. Best practice for biodiversity data management and publication. Biological Conservation. 173: 68–73.

[bib9] Cox E, Tsuchiya MT, Ciufo S, Torcivia J, Falk R, Anderson WR, O’Leary NA. 2025. NCBI taxonomy: Enhanced access via NCBI datasets. Nucleic Acids Research. 53:D1711–D1715. 39470745 10.1093/nar/gkae967 PMC11701650

[bib10] Del Hoyo J, Collar NJ. 2014. The HBW‒BirdLife International Illustrated Checklist of The Birds of The World, vol. 1: Non-Passerines. Lynx Edicions, Barcelona.

[bib11] Del Hoyo J, Collar NJ. 2016. The HBW‒BirdLife International Illustrated Checklist of The Birds of The World, vol. 2: Passerines. Barcelona: Lynx Edicions.

[bib12] Dobbie MJ, Dail D. 2013. Robustness and sensitivity of weighting and aggregation in constructing composite indices. Ecological Indicators. 29: 270–277.

[bib13] Döring M, Jeppesen T, Bánki O. 2022. Introducing ChecklistBank: An index and repository for taxonomic data. Biodiversity Information Science and Standards. 6: e93938.

[bib14] Espeland W, Stevens M. 2008. A sociology of quantification. European Journal of Sociology. 49: 401–436. 10.1017/S0003975609000150.

[bib15] Evenhuis NL, Pape T, eds. 2024. Systema Dipterorum, vers. 5.1. Diptera. http://diptera.org/on.

[bib16] Farjon A, Gardner M, Thomas P. 2015. Conifer Database.Article 45in Bánki O et al. Catalogue of Life Checklist. Catalogue of Life. https://www.catalogueoflife.org/annual-checklist/2015/details/database/id/45.

[bib17] Faurby S, Eiserhardt WL, Svenning J-C. 2016. Strong effect of variation in taxonomic opinion on diversification analyses. Methods in Ecology and Evolution. 7: 4–13.

[bib18] Feng X, Enquist BJ, Park DS, Boyle B, Breshears DD, Gallagher RV, López-Hoffman L. 2022. A review of the heterogeneous landscape of biodiversity databases: Opportunities and challenges for a synthesized biodiversity knowledge base. Global Ecology and Biogeography. 31: 1242–1260.

[bib19] Franceschini F, Galetto M, Maisano D. 2006. Classification of performance and quality indicators in manufacturing. International Journal of Services and Operations Management. 2: 294–311.

[bib20] Franceschini F, Galetto M, Maisano D. 2019. Designing Performance Measurement Systems. Theory and Practice of Key Performance Indicators. Springer.

[bib21] Franz N, Gilbert E, Ludäscher B, Weakley A. 2016. Controlling the taxonomic variable: Taxonomic concept resolution for a southeastern United States herbarium portal. Research Ideas and Outcomes. 2: e10610. doi:10.3897/rio.2.e10610.

[bib22] Franz NM, Peet RK, Weakley AS. 2008. On the use of taxonomic concepts in support of biodiversity research and taxonomy. Systematics Association Special Volume. 76: 63–86.

[bib23] Fricke R, Eschmeyer WN, Van der Laan R, eds. 2025. Eschmeyer’s Catalog of Fishes: Genera, Species, References. California Academy of Sciences. https://www.calacademy.org/scientists/projects/eschmeyers-catalog-of-fishes.

[bib71_763_143425] Froese R, Pauly D. (eds.). 2024. FishBase version 02/2024. http://www.fishbase.org. 9 April 2024.

[bib25] Garnett ST et al. 2025. Measuring the quality of species list governance. BioScience. 75: present issue. 10.1093/biosci/biaf071 PMC13032865 41907827

[bib70_568_144525] Gómez-Limón JA, Sanchez-Fernandez G. 2010. Empirical evaluation of agricultural sustainability using composite indicators. Ecological Economics. 69: 1062–1075.

[bib26] Gramsci A. 1982. La Città Futura: 1917–1918. Guilio Einaudi.

[bib32] [IUCN] International Union for Conservation of Nature . 2024. The IUCN Red List of Threatened Species, vers. 2023-1. IUCN. www.iucnredlist.org.

[bib27] Hauser J, Katz G. 1998. Metrics: You are what you measure!. European Management Journal. 16: 517–528.

[bib28] Hobern D et al. 2021. Towards a global list of accepted species VI: The Catalogue of Life checklist. Organisms Diversity and Evolution. 21: 677–690.

[bib29] Horbach SS, Halffman WW. 2018. The changing forms and expectations of peer review. Research Integrity and Peer Review. 3: 1–15. 30250752 10.1186/s41073-018-0051-5 PMC6146676

[bib30] Huber BA, Szymański H, Bennett-West A. 2024. Progress or burden? Formal description of every apparently new species available in collections is neither necessary nor useful. ZooKeys. 1214: 77. 39391536 10.3897/zookeys.1214.130592 PMC11462076

[bib31] Hubert N, Hanner R. 2015. DNA barcoding, species delineation and taxonomy: A historical perspective. DNA Barcodes. 3: 44–58.

[bib33] Kar AK, Varsha PS, Rajan S. 2023. Unravelling the impact of generative artificial intelligence (GAI) in industrial applications: A review of scientific and grey literature. Global Journal of Flexible Systems Management. 24: 659–689.

[bib34] Kitchener A, Hoffmann M, Yamaguchi N, Breitenmoser-Würsten C, Wilting A. 2022. A system for designating taxonomic certainty in mammals and other taxa. Mammalian Biology. 102: 251–261.

[bib35] Kroh A, Mooi R. 2025. The World Echinoidea Database, vers. 3.0. World Echinoidea Database. www.marinespecies.org/echinoidea.

[bib36] Legume Phylogeny Working Group . 2024. Legume Data Portal. Legume Phylogeny Working Group. www.legumedata.org.

[bib37] Lessa T, Stropp J, Hortal J, Ladle RJ. 2024. How taxonomic change influences forecasts of the linnean shortfall (and what we can do about it)?. Journal of Biogeography. 51: 1365–1373.

[bib38] Lien AM et al. 2023. Widespread support for a global species list with a formal governance system. Proceedings of the National Academy of Sciences. 120: e2306899120. 10.1073/pnas.2306899120 PMC10636331 37903262

[bib39] Martin TE, Bennett GC, Fairbairn A, Mooers AO. 2023. "Lost" taxa and their conservation implications. Animal Conservation. 26: 14–24.

[bib40] Millett P, Alexanian T, Brink KR, Carter SR, Diggans J, Palmer MJ, Ritterson R, Sandbrink JB, Wheeler NE. 2023. Beyond biosecurity by taxonomic lists: Lessons, challenges, and opportunities. Health Security. 21: 521–529. 37856148 10.1089/hs.2022.0109 PMC10733751

[bib41] Minelli A. 2020. Taxonomy needs pluralism, but a controlled and manageable one. Megataxa. 1: 9–18.

[bib42] Munda G, Nardo M. 2005. Constructing Consistent Composite Indicators: The Issue of Weights. European Commission, Joint Research Centre, Institute for the Protection and Security of the Citizen.

[bib43] [NCBI] National Center for Biotechnology Information . 2025. National Library of Medicine National Center for Biotechnology Information. NCBI. www.ncbi.nlm.nih.gov.

[bib44] Norton P, Hughes M. 2017. Public Consultation and Community Involvement in Planning: A Twenty-first Century Guide. Routledge.

[bib45] Noy S, Zhang W. 2023. Experimental evidence on the productivity effects of generative artificial intelligence. Science. 381: 187–192. 37440646 10.1126/science.adh2586

[bib46] Palmer MW, McGlinn DJ, Fridley JD. 2008. Artifacts and artifictions in biodiversity research. Folia Geobotanica. 43: 245–257.

[bib47] Palmer MW, Richardson JC. 2012. Biodiversity data in the Information age: Do 21st century floras make the grade?. Castanea. 77: 46–59. 10.2179/11-035.

[bib48] Palmer MW, Wade GL, Neal PR. 1995. Standards for the writing of floras. BioScience. 45: 339–345.

[bib49] Penev L et al. 2016. A common registration-to-publication automated pipeline for nomenclatural acts for higher plants (International Plant Names Index, IPNI), fungi (Index Fungorum, MycoBank) and animals (ZooBank). ZooKeys. 550: 233. 10.3897/zookeys.550.9551 PMC4741224 26877662

[bib50] Phipps A. 2019. Decolonising Multilingualism: Struggles to Decreate. Multilingual Matters.

[bib51] Pühringer F, Kallies A. 2004. Provisional checklist of the Sesiidae of the world (Lepidoptera: Ditrysia). Mitteilungen der Entomologischen Arbeitsgemeinschaft Salzkammergut. 4: 1–85.

[bib52] Pyle RL et al. 2021. Towards a global list of accepted species V: The devil is in the detail. Organisms Diversity and Evolution. 21: 657–675.

[bib53] Pyle RL, Michel E. 2008. ZooBank: Developing a nomenclatural tool for unifying 250 years of biological information. Zootaxa. 1950: 39–50.

[bib56] Ruggiero MA, Gordon DP, Orrell TM, Bailly N, Bourgoin T, Brusca RC, Cavalier-Smith T, Guiry MD, Kirk PM. 2015. A higher level classification of all living organisms. PLOS ONE. 10: e011. 10.1371/journal.pone.0119248. PMC4418965 25923521

[bib58] [SANBI] South African National Biodiversity Institute . 2024. The South African National Plant Checklist: 2024 Official Yearly Release. SANBI. http://hdl.handle.net/20.500.12143/6880.2.

[bib57] Saisana M, Tarantola S. 2002. State-of-the-Art Report on Current Methodologies and Practices for Composite Indicator Development. European Commission, Joint Research Centre, Institute for the Protection and the Security of the Citizen, Technological and Economic Risk Management Unit.

[bib59] Sandall EL et al. 2023. A globally integrated structure of taxonomy to support biodiversity science and conservation. Trends in Ecology and Evolution. 38:1143–1153. 37684131 10.1016/j.tree.2023.08.004

[bib60] Sinclair R. 2020. Un-settling species concepts through indigenous knowledge: Implications for ethics and science. Environmental Ethics. 42: 313–334.

[bib61] Tennant JP et al. 2017. A multi-disciplinary perspective on emergent and future innovations in peer review. F1000Research. 6: 1151. 29188015 10.12688/f1000research.12037.1 PMC5686505

[bib62] Thomson SA et al. 2021. Towards a global list of accepted species II: Consequences of inadequate taxonomic list governance. Organisms Diversity and Evolution. 21: 623–630.

[bib63] Torres-Mejía M, Beltrán N, Llano S. 2016. Listas de Especies: Lineamientos Conceptuales y Metodológicos para Su Consolidación en Colombia. Colombia, Bogota: Sistema de Información sobre Biodiversidad de.

[bib55] Turtle Extinction Working Group . 2015. Turtles and Tortoises of the World during the Rise and Global Spread of Humanity: First Checklist and Review of Extinct Pleistocene and Holocene Chelonians. Chelonian Research Monographs. https://iucntftsg.org/wpcontent/uploads/file/Accounts/crm_5_000e_fossil_checklist_v1_2015.pdf.

[bib54] Turtle Taxonomy Working Group . 2021. Turtles of the World: Annotated Checklist and Atlas of Taxonomy, Synonymy, Distribution, and Conservation Status, 9th ed. Chelonian Research Monographs. 10.3854/crm.8.checklist.atlas.v9.2021.

[bib64] Ware M. 2008. Peer review: Benefits, perceptions, and alternatives. PRC Summary Papers. 4: 2–22.

[bib65] Winston JE. 2018. Twenty-first century biological nomenclature: The enduring power of names. Integrative and Comparative Biology. 58: 1122–1131. 10.1093/icb/icy060. 30113637

[bib66] World Flora Online . 2024a. Conifers. The World Flora Online Project. https://about.worldfloraonline.org/tens/conifers.

[bib67] World Flora Online . 2024b. About the World Flora Online Project. World Flora Online Project. https://about.worldfloraonline.org

[bib68] Wrankmore E, Krieger J, Govaerts R, Hartley H. 2022. Demonstration of the new IPNI (International Plant Names Index) registration system. Biodiversity Information Science and Standards. 6: e91371.

