# Supplementary material for: Measuring the Quality of Species List Contents
Source: Bioscience. 2026 Jan 28;76(3):269–83. doi: 10.1093/biosci/biaf191 (PMC13032866; doi:10.1093/biosci/biaf191)
Supplement: biaf191_Supplemental_Files [file biaf191_supplemental_files.zip › List_Contents_Supplementary_Table 1_updated_R2.docx]

**Supplementary Table 1.** Desirable details for species lists (from Pyle et al. 2021). Those marked with an asterisk were used to develop content quality indicators.

| **List detail** | **Question on detail to be answered** |
| --- | --- |
| 1. Higher taxa* | Does list include higher-rank taxa (e.g., families, orders, classes, phyla, kingdoms) and what granularity (e.g., subfamilies, tribes, etc.)? |
| 2. Level of confidence* | Does list include *incertae sedis* and provisional names? |
| 3. Names outside the scope of Code-regulated nomenclature* | Does list include, e.g., cultivars, temporary (e.g., “sp,1234”), unranked names (e.g., Bilateria)? |
| 4. Extinct taxa based on fossils* | Does list include taxa known only from fossils with no live individuals known before 1500 AD? |
| 5. Taxa lacking scientific names* | Does list include un-named nodes of a hypothesized phylogeny? |
| 6. Unique persistent identifier* | Is each item on list branded with a globally unique identifier with unambiguous and persistent mechanisms to resolve or retrieve metadata? |
| 7. Version history* | Does each entry have a robust “audit trail” for its metadata? |
| 8. Label* | Does each entry have a unique label – either a scientific name compliant with the relevant nomenclatural Code or a widely recognized standardized informal taxon label? |
| 9. Nomenclatural authorship* | Does each entry include nomenclatural authorship, formatted in accordance with the relevant nomenclatural Code? |
| 10. Source* | Does each entry have a link or textual description of the database or working group that provided the entry with properties of the source or a link to source metadata? |
| 11. Nomenclatural Code* | Does list specify the nomenclatural Code (or Codes for ambiregnal taxa) for each scientific name? |
| 12. Original ranks and combinations* | Do names below the rank of genus include the basionym combination, or original rank for names treated as subgenera/subspecies that were originally established as full genera/species, and vice versa? |
| 13. Original literature citation* | Is the basionym citation included as a full textual citation or with a standard unique identifier (e.g., DOI)? |
| 14. Accepted status treatment citation* | Is each entry linked to a treatment representing the current/accepted taxonomic status of the entry? |
| 15. Confidence* | Is there an indication of confidence in the current status of the entry in e.g., conformity with relevant Code, acceptance by relevant taxonomic community, taxonomic rank, etc.? |
| 16. Synonymy* | Are all heterotypic and homotypic synonyms, as well as misspellings and other orthographic variants included with each entry? |
| 17. Classification* | Can all taxa ranked below genus be linked to a genus and can all genera be linked to higher level ranks? |
| 18. Geographical distribution* | Are entries associated with geographical information? |
| 19. Images* | Are entries associated with images? |
| 20. Annotations* | Are entries annotated with additional information? |
| 21. Associated vernacular names | Are entries associated with vernacular names? |
| 22. Type specimen(s)* | Are entries associated with information on type specimen(s)? |
| 23. Character diagnoses* | Are entries associated with diagnostic characters (e.g., morphological, biochemical, genetic)? |
| 24. Species concept | Are entries associated with a species concept? |
| 25. Creating and maintaining the global list | Are there processes by which taxonomic communities evaluate the accuracy and legitimacy of list content? |
| 26. Identifying and completing the gaps | Does the list have processes for identifying and filling gaps? |
| 27. Newly described species and new taxonomic arrangements | Does the list have processes for including newly described taxa and revisions? |
| 28. Peer-review | Is the list subject to peer-review? |
| 29. Arbitrating taxonomic disagreements | Does the list have a process for arbitrating disputes? |
| 30. The local and the global | Do global lists have processes for accommodating local perspectives? |
| 31. Accessing and citing the global list | Is the list freely available under a creative commons license? |
