# Supplementary material for: Measuring the Quality of Species List Contents
Source: Bioscience. 2026 Jan 28;76(3):269–83. doi: 10.1093/biosci/biaf191 (PMC13032866; doi:10.1093/biosci/biaf191)
Supplement: biaf191_Supplemental_Files [file biaf191_supplemental_files.zip › List_Contents_Supplementary_Table 2_updated_R2.docx]

**Supplementary Table 2. Normalised scores (out of 10) for the desirability of an attribute of a taxonomic list (based on Lien et al. 2023)**

| **Content indicator** | **Scores** |
| --- | --- |
| C2. Completeness: How undesirable or desirable is it that datasets are complete or accompanied by a description of how gaps will be filled for each taxon on a global species list? | 8.7 |
| C3. Recently extinct taxa: How undesirable or desirable is the inclusion of recently extinct taxa for each taxon on a global species list? | 7.9 |
| C4. Fossils: How undesirable or desirable is the inclusion of fossil taxa for each taxon on a global species list? | 5.4 |
| C5. Non-Code-regulated names: How undesirable or desirable is the inclusion of non-Linnaean names for each taxon on a global species list? | 3.0 |
| C7. Nomenclatural code: How undesirable or desirable is the inclusion of the name of the relevant nomenclatural code for each taxon on a global species list? | 6.6 |
| C8. Classification detail: How undesirable or desirable is classification above the genus-level for each taxon on a global species list? | 8.7 |
| C9. Unique, persistent identifiers: How undesirable or desirable is the inclusion of a unique, persistent identifier consistent with biodiversity information standards and practices for each taxon on a global species list? | 8.5 |
| C10. Nomenclatural authority: How undesirable or desirable is the inclusion of the nomenclatural author of the taxon, with date for each taxon on a global species list? | 9.5 |
| C11. Treatment authority: How undesirable or desirable is the inclusion of information on treatment author of the taxon, with citation for each taxon on a global species list? | 8.7 |
| C13. Original ranks and combinations: How undesirable or desirable is the inclusion of type-specimen information for each taxon on a global species list? | 9.1 |
| C14. Original literature citation: How undesirable or desirable is the inclusion of type-specimen information for each taxon on a global species list? | 9.1 |
| C17. Homotypic synonyms: How undesirable or desirable is the inclusion of synonyms of taxa for each taxon on a global species list? | 10.0 |
| C18. Heterotypic synonyms: How undesirable or desirable is the inclusion of synonyms of taxa for each taxon on a global species list? | 10.0 |
| C20. Documentation of change: How undesirable or desirable is the inclusion of version history for each taxon on a global species list? | 8.8 |
| C21. Geographical distribution: How undesirable or desirable is the inclusion of information on geographical distribution of taxa for each taxon on a global species list? | 9.9 |
| C22. Images: How undesirable or desirable is the inclusion of images for each taxon on a global species list? | 8.7 |
| C24. Additional information: How undesirable or desirable is a system of monitored and moderated annotations to enable user engagement for each taxon on a global species list? | 7.1 |
| The following indicators were not included in the questionnaire of Lien et al. (2023) so were allocated the average score for all indicators (8.2) for the purposes of weighting list scores. | |
| C1. Scope: Weighting score calculated from average of other scores. | |
| C6. Inclusion: Not included. Weighting score calculated from average of other scores. | |
| C12. Source of name: Not included. Weighting score calculated from average of other scores. | |
| C15. Citation completeness: Not included. Weighting score calculated from average of other scores. | |
| C16. Current status literature citation: Not included. Weighting score calculated from average of other scores. | |
| C19. Confidence in taxonomic status: Not included. Weighting score calculated from average of other scores. | |
| C23. Genetic data: Not included. Weighting score calculated from average of other scores. | |
| C23. Genetic data: Not included. Weighting score calculated from average of other scores. | |
