# Supplementary material for: Measuring the Quality of Species List Contents
Source: Bioscience. 2026 Jan 28;76(3):269–83. doi: 10.1093/biosci/biaf191 (PMC13032866; doi:10.1093/biosci/biaf191)
Supplement: biaf191_Supplemental_Files [file biaf191_supplemental_files.zip › List_Contents_Supplementary_Table 3_updated_R2.docx]

**Supplementary Table 3. Scores for contents indicators for 16 taxonomic lists**

| Content quality measure (text) | 1. Conifera (2024) | 2. Conifera (2014) | 3. Leguminosae | 4. South African Plants | 5. Orthoptera | 6. Diptera | 7. Sesiidae | 8. Coleoptera (genera) | 9. Fossil Cirripedia | 10. Echinodermata | 11. Fossil Echinodermata | 12. Fishbase | 13. Catalog of Fishes | 14. Testudines | 15. Fossil Testudines | 16. Aves |
| --- | --- | --- | --- | --- | --- | --- | --- | --- | --- | --- | --- | --- | --- | --- | --- | --- |
| Scope | 5 | 5 | 5 | 5 | 5 | 5 | 5 | 5 | 5 | 5 | 5 | 5 | 5 | 5 | 5 | 5 |
| Completeness | 5 | 4 | 5 | 5 | 5 | 4 | 5 | 5 | 4 | 5 | 5 | 5 | 5 | 5 | 5 | 4 |
| Recently extinct taxa | 4 | 4 | 4 | 4 | 4 | 5 | 5 | 5 | 5 | 5 | 5 | 4 | 4 | 4 | 5 | 5 |
| Fossils | 0 | 0 | 0 | 0 | 5 | 5 | 0 | 5 | 5 | 5 | 5 | 5 | 5 | 5 | 5 | 0 |
| Non-Code-regulated names | 5 | 5 | 4 | 3 | 5 | 3 | 5 | 5 | 5 | 5 | 5 | 4 | 4 | 5 | 5 | 5 |
| Pre-listing review | 0 | 0 | 0 | 5 | 5 | 5 | 0 | 5 | 0 | 0 | 0 | 0 | 0 | 5 | 5 | 5 |
| Nomenclatural code | 5 | 4 | 4 | 5 | 5 | 4 | 4 | 5 | 4 | 4 | 4 | 4 | 4 | 5 | 4 | 4 |
| Classification detail | 5 | 4 | 5 | 5 | 5 | 5 | 5 | 5 | 5 | 5 | 5 | 5 | 5 | 5 | 5 | 5 |
| Unique, persistent identifier | 0 | 0 | 5 | 5 | 5 | 5 | 0 | 5 | 0 | 5 | 5 | 5 | 5 | 5 | 5 | 5 |
| Nomenclatural authority | 5 | 5 | 5 | 5 | 5 | 5 | 5 | 5 | 3 | 5 | 5 | 5 | 5 | 5 | 5 | 5 |
| Treatment authority | 5 | 3 | 5 | 5 | 5 | 3 | 5 | 5 | 5 | 5 | 5 | 5 | 5 | 5 | 5 | 5 |
| Source of name | 5 | 1 | 0 | 5 | 5 | 3 | 5 | 5 | 5 | 5 | 5 | 5 | 5 | 5 | 5 | 5 |
| Original ranks and combinations | 5 | 5 | 5 | 5 | 5 | 5 | 1 | 5 | 5 | 5 | 5 | 5 | 5 | 5 | 5 | 5 |
| Original literature citation | 5 | 5 | 5 | 3 | 5 | 5 | 5 | 5 | 5 | 5 | 5 | 5 | 5 | 5 | 5 | 3 |
| Citation completeness | 2 | 4 | 5 | 2 | 5 | 2 | 0 | 2 | 1 | 5 | 5 | 5 | 5 | 5 | 2 | 2 |
| Current status literature citation | 5 | 3 | 0 | 5 | 5 | 3 | 3 | 3 | 4 | 5 | 5 | 5 | 5 | 5 | 3 | 3 |
| Homotypic synonyms | 5 | 3 | 5 | 4 | 5 | 5 | 1 | 5 | 5 | 5 | 5 | 5 | 5 | 5 | 4 | 5 |
| Heterotypic synonyms | 5 | 3 | 5 | 4 | 5 | 5 | 5 | 5 | 5 | 5 | 5 | 5 | 5 | 5 | 4 | 5 |
| Confidence in taxonomic rank and placement | 0 | 0 | 0 | 0 | 0 | 5 | 0 | 0 | 0 | 0 | 0 | 0 | 0 | 0 | 0 | 5 |
| Documentation of change | 1 | 0 | 3 | 5 | 0 | 0 | 3 | 0 | 3 | 0 | 0 | 0 | 0 | 0 | 1 | 3 |
| Geographical distribution | 3 | 3 | 3 | 3 | 5 | 3 | 5 | 5 | 3 | 5 | 5 | 3 | 3 | 5 | 3 | 5 |
| Images | 0 | 0 | 0 | 3 | 5 | 0 | 0 | 5 | 0 | 5 | 5 | 0 | 0 | 5 | 5 | 5 |
| Genetic data | 0 | 0 | 0 | 0 | 0 | 0 | 0 | 0 | 5 | 0 | 5 | 0 | 0 | 0 | 5 | 0 |
| Additional information | 0 | 0 | 0 | 3 | 5 | 0 | 0 | 5 | 0 | 5 | 5 | 0 | 0 | 5 | 5 | 5 |
