# Supplementary material for: Measuring the Quality of Species List Contents
Source: Bioscience. 2026 Jan 28;76(3):269–83. doi: 10.1093/biosci/biaf191 (PMC13032866; doi:10.1093/biosci/biaf191)
Supplement: biaf191_Supplemental_Files [file biaf191_supplemental_files.zip › List_Contents_Supplementary_Table 4_updated_R2.docx]

**Supplementary Table 4.** The potential for automation of indicators using ChecklistBank.

| **Indicator** | **Potential for automation using ChecklistBank** | | **Indicator** | **Potential for automation using ChecklistBank** |
| --- | --- | --- | --- | --- |
| Scope | Metadata |  | Original ranks and combinations | Computable |
| Completeness | Computable, particularly if "known taxa" is provided in metadata | | Original literature citation | Computable if authorship is given |
| Recently extinct taxa | Metadata |  | Citation completeness | Potentially computable if original citation is defined as the publication that established the exact combination |
| Fossils | Metadata |  | Current status literature citation | Computable |
| Non-Code-regulated names | Metadata |  | Homotypic synonyms | Metadata |
| Pre-listing review | Metadata |  | Heterotypic synonyms | Metadata |
| Nomenclatural code | Metadata |  | Confidence in taxonomic rank and placement | Metadata |
| Classification detail | Either in metadata or computable |  | Documentation of change | Metadata |
| Unique, persistent identifier | Computable |  | Geographical distribution | Metadata |
| Nomenclatural authority | Computable |  | Images | Metadata |
| Treatment authority | Either in metadata, in which the list can define itself as the treatment, or computable | | Genetic data | Metadata |
| Source of name | Computable |  | Additional information | Metadata |
